# Perceived Discrimination and Self-Rated Health in South Korea: A Nationally Representative Survey

**DOI:** 10.1371/journal.pone.0030501

**Published:** 2012-01-17

**Authors:** Seung-Sup Kim, David R. Williams

**Affiliations:** 1 Department of Environmental and Occupational Health, The George Washington University School of Public Health and Health Services, Washington, D.C., United States of America; 2 Department of Society, Human Development, and Health, Harvard School of Public Health, Boston, Massachusetts, United States of America; Wayne State University, United States of America

## Abstract

**Background:**

There is mounting evidence that discriminatory experiences can harm health. However, previous research has mainly focused on the health effects of racial discrimination in U.S. or European countries although there is pervasive discrimination by gender, age, education and other factors in Asian countries.

**Methods:**

We analyzed the data from the 7th wave of Korean Labor and Income Panel Study to investigate the association between perceived discriminatory experience and poor self-rated health in South Korea. Perceived discriminatory experiences were measured in eight situations through a modified Experience of Discrimination questionnaire. In each of eight situations, the lifetime prevalence of perceived discriminatory experience was compared between men and women and the main causes of those experiences were identified separately by gender. After adjusting for potential confounders, we examined the association between perceived discriminatory experience and poor self-rated health in each of eight social situations and also checked the association using the number of situations of perceived discriminatory experiences.

**Results:**

For both men and women, education level and age were the main sources of work-related perceived discriminatory experiences. Gender was one of the main causes among women across eight situations and more than 90% of women reported their gender as a main cause of discriminatory experience in getting higher education and at home. Discriminatory experiences in four situations were positively associated with poor self-rated health. The odds ratio for poor self-rated health for those exposed to one, two, three or four or more social situations of perceived discrimination were respectively 1.06 (95% CI : 0.87–1.29), 1.15 (95% CI : 0.96–1.55), 1.59 (95% CI : 1.19–2.14), and 1.78 (95% CI :1.26–2.51).

**Conclusion:**

There is consistent association between perceived discriminatory experience and poor self-rated health across eight social situations in South Korea.

## Introduction

There is growing scientific interest in the multiple ways in which discrimination can harm health. Studies in several national and cultural contexts have shown that there is a strong and consistent pattern in which self-reports of discrimination are positively associated with indicators of poor physical and mental health such as hypertension, heart disease and depression as well as indicators of high-risk health behaviors [1]–[3]. The previous literature has emphasized the health effects of discrimination based on race or ethnicity [4]–[11].

However, a growing body of evidence suggests that other types of self-reported discrimination, based on other factors, such as an individual's gender, weight, sexual orientation or age, can also be predictive of poor health status. This is evident in U.S. data and also in studies from other countries [1]. For example, analyses from the Whitehall Study in the UK have found that non-racial discrimination was an independent risk factor for poor health outcomes. *Ferrie and the colleagues* found that unfair treatment by supervisors was associated with incident psychiatric disorders [12]. Similarly, prospective analyses from Whitehall found that self-reported unfairness was related to both incident coronary events and metabolic syndrome among white-collar British civil servants [13], [14]. Studies in South Africa, have also found that non-racial as well as racial discrimination was associated with increased risks of mental health problems, even after adjustment for socio-demographic factors and other stressors [15], [16].

However, there have been few empirical studies of the prevalence of perceived discrimination and its potential effects on health in Asian countries, even though these countries have pervasive discrimination based on gender, age, education level, birth region, sexual orientation and race or ethnicity [17]–[20]. Prior studies in Asian countries have focused on the health effects of discriminatory experiences of specific stigmatized groups such as rural to urban migrants in China [21], Japanese Brazilians in Japan[19], and young Mainland Chinese immigrants in Hong Kong [20]. However, we are unaware of any research on the health effects of perceived discrimination in a general population of an Asian country.

South Korea's history of strong patriarchal social traditions [22] and the Confucian ideology of male superiority [23] is believed to contribute to systemic patterns of discrimination that women encounter in multiple settings. At home, they are required to carry out most domestic responsibilities and face a high level of sex-selective abortion [23]–[25]. At work, female workers are more likely than their male peers (63.5% versus 39.7% in 2010) to work in non-permanent jobs which are low paid and unstable [22], [26]. Compared with men, women in South Korea also have lower socioeconomic status and a higher prevalence of depression and chronic diseases [27]. However, to date, no research in South Korea has examined how discriminatory experience is associated with health outcome in women, and how this association differs from that in men.

This paper explored the association between perceived discriminatory experiences and poor self-rated health and whether this association is modified by gender using a nationally representative survey from South Korea. Specifically, our research sought answer to the following questions:

1. What is the prevalence of lifetime discriminatory experiences in South Korea? Does it differ by gender?
2. What are the main sources of attribution for perceived discrimination in South Korea? Do these differ by gender?
3. How are experiences of discrimination related to self-rated health? Does this association vary by the social context in which the experience occurs? Does the association reflect a dose-response pattern?

## Methods

### Data

The Korean Labor and Income Panel Study (hereafter KLIPS) is a nationally representative panel survey of the urban population in South Korea. The first survey was launched in 1998, and data have been collected yearly since then. The data were collected through in-person interviews by trained personnel. The KLIPS recruited 5000 households in urban areas, using two stage stratified cluster sampling at the baseline. To date, data from the first to 11^th^ waves (1998–2008) have been publicly released [http://www.kli.re.kr/]. The 7^th^ wave (2004) of the survey included questions on experiences of discrimination, and data from this wave were used for this cross-sectional analysis.

### Measures

Perceived discrimination was measured using a modified version of the Experience of Discrimination (EOD) questionnaire [28], which asked participants whether they had “ever experienced discrimination” in eight specific situations: in getting hired, income, training, promotion, fired, getting higher education, at home, and in general social activities. For each question, participants answered “Yes”, “No”, or “Not Applicable”. Those responding “Yes”, were asked to report the main reason why they thought they had been discriminated against. These included “gender”, “age”, “education level”, “disability”, birth region”, and “other”. Certain birth regions in South Korea, such as Cholla province including Chollanam-do, Chollabuk-do and Gwangju have historically been politically and economically isolated and stigmatized [29], [30]. Participants were allowed to report multiple causes of perceived discriminatory experiences.

The health outcome, self-rated health, was measured with the question “How would you rate your overall health?” Responses ranging from ‘excellent’ (1) to ‘very poor’ (5) were dichotomized into good health (0, for responses 1–3) and poor health (1, for responses 4–5). Self-rated health has been shown to be predictive of objective health outcomes [31], [32]. For example, a review of 27 empirical studies found self-rated health to be an independent predictor of mortality even after adjusting for potential confounders, including numerous specific health-status indicators [33].

Several potential confounders were included in the study. Age was included as a continuous variable. Marital status was categorized into never, currently, and previously married, with the currently married as the reference group. Education was coded into four categories: junior high or less (reference group), high school graduate, college graduate, and university graduate or more. We adjusted for income and employment status because they are known correlates of experiences of discrimination and of health outcomes. An equivalized monthly household income was calculated by summing all sources of income including earnings, interest, rents and dividends and dividing it by the square root of the number of household members. The resulting income measure was categorized into four quartiles using the lowest quartile as a reference group. Occupation was categorized into five mutually exclusive categories: “precarious worker”, “non-precarious worker”, “employer”, “full-time student” and “unemployed” (included full-time housewives). Each occupational category was coded as a dummy variable ( = 1) with “unemployed” used as the reference group ( = 0). Precarious workers were defined as wage workers employed temporarily, daily, or part-time, while all other wage workers were defined as non-precarious. Previous studies show that precarious workers are disadvantaged compared to non-precarious workers in terms of wages, social benefits, labor union membership, and health status in South Korea [34], [35].

### Data Analyses

We first estimated the gender-specific prevalence of each of the eight specific perceived discriminatory experiences, after excluding the respondents who answered ‘Not applicable’. We also examined by gender the proportion of participants reporting the six most common sources of perceived discrimination (i.e., gender, age, education level, disability, birth region, and other). Participants were allowed to report multiple causes of their perceived discriminatory experiences.

All analyses were performed using STATA/SE version 11.0 (StataCorp, College Station, TX). Multivariate logistic regression was used to examine associations between self-reported lifetime experiences of discrimination and poor self-rated health controlling for multiple covariates in each of eight situations after excluding the participants answering “Not Applicable” in each situation. In the fully adjusted model, we tested whether the health effect of perceived discriminatory experiences was modified by gender in each of the eight situations. Furthermore, in order to check for a dose-response relationship between perceived discriminatory experiences and self-rated health, the discrimination score was coded 0 through 4, corresponding to whether the participants had experienced discrimination in 0, 1, 2, 3, or 4 or more situations. There were respondents (n = 4,249) who answered “Yes” or “No” for at least one situation and “Not Applicable” for other situations, they are included in calculating the discrimination score, assuming that had not experienced discrimination in the situations that they answered “Not Applicable”. However, a dummy variable was created that contrasted respondents, who answered “Not Applicable” for all eight situations (n = 584) to those who did not. This variable was included in the model. Because participants belonging to the same family are likely to have similar exposures and outcomes (4746 participants had family members in the sample), the Huber-White sandwich estimator were used to calculate odds ratio confidence intervals robust to within-family clustering [36], [37].

### Ethics

The KLIPS is the publicly released dataset which can be downloaded from the website of Korea Labor Institute (http://www.kli.re.kr/). So we do not need an informed consent to use this dataset. This research received IRB exemption from Office of Human Research Administration at the Harvard School of Public Health.

## Results

Table 1 presents the estimated prevalence of the lifetime prevalence of discrimination by sociodemographic characteristics. Women (vs. men), those with lower education (vs. higher education), non-precarious workers (vs. precarious workers and employers), the previously married (vs. those never married, the currently married) are significantly more likely to have experienced discrimination.

**Table 1:** Distribution of Study Population and Prevalence of Lifetime Perceived Discrimination, South Korea, 2004 (n = 11,544).

	Distribution (%)	Lifetime prevalence of any reported discrimination (%)	P-value^a^
Sex			
Male	48.2	20.0	
Female	51.8	22.1	0.004
Age (years old)			
16–25	15.8	11.1	
25–34	21.7	22.4	
35–44	20.6	23.0	
45–54	17.3	23.4	
55–64	11.9	24.8	
65–	12.7	21.8	0.001
Education			
Junior high or less	30.5	26.3	
High school graduate	36.6	20.9	
College graduate	11.2	21.7	
University and more	21.7	13.7	<0.001
Household income			
1Q-	24.2	26.4	
2Q–3Q	25.4	23.2	
3Q–4Q	25.3	20.6	
4Q-	25.1	14.4	<0.001
Marriage			
Never married	27.0	10.6	
Previously married	21.6	62.7	
Current married	17.6	26.6	<0.001
Occupation			
Unemployed	39.0	22.6	
Full time student	11.1	4.05	
Precarious employment	8.6	38.4	
Non-precarious employment	27.9	23.4	
Employer	13.4	14.9	<0.001

a P-value : Chi-square test about the difference of lifetime prevalence of perceived discriminatory experience across different socio-demographic groups.

The distribution of various types of discrimination and the reported reasons for discrimination, for both men and women, are reported in Table 2. Men report higher prevalence of discriminatory experiences in getting promoted than women but women report higher prevalence of discrimination in education and at home than men. There was no gender difference in perceived discrimination in other work-related situations and in general social activities. Gender, education level and age were the most frequently reported reasons for discriminatory experiences across the eight situations. The most common reason reported by men was education level, followed by age. Attribution to other reasons was high for some specific types of discrimination. Among men, disability was the most common reason for perceived discrimination in getting higher education, while birth region was the most frequently mentioned in getting promoted. For both men and women, age was the most common reason reported for discrimination in getting fired. Among women, gender was the most common reason for perceived discrimination with more than 35% of women reporting gender as a cause of their experience across all the types of discrimination. Regarding discriminatory experiences outside of the workplace, more than 90% of women reported their gender as the source of discriminatory experiences in getting higher education and at home. Discriminatory experiences because of education level and age were frequently reported by women for work-related discriminatory experiences.

**Table 2:** Prevalence of Main Reasons for Perceived Discriminatory Experience After Stratified by Gender, South Korea, 2004.

Men (n = 5,561)	Prevalence^a^		Distribution of main reasons for discriminatory experience^b^ (%)						
	No. of respondents^c^	N (%)	sex	education	age	disability	birth region	others	not reply
Hired	4,393	809 (18.4)	3.2	62.1	41.5	5.2	4.2	5.8	0
Income	4,424	563 (12.7)	5.2	75.0	32.6	3.7	1.9	0	4.8
Training	4,115	84 (2.0)	5.0	62.5	20.0	18.8	5.0	0	4.8
Promotion	4,024	253 (6.3)**^d^	3.9	82.3	14.7	4.3	13.9	0	8.7
Fired	4,103	101 (2.5)	1.1	35.2	58.0	12.5	6.8	0	12.9
Education	5,091	19 (0.4)*** d	11.1	16.7	16.7	55.6	0	0	5.3
At home	5,229	27 (0.5)*** d	37.5	25.0	8.3	29.2	0	0	11.1
Societal activities	5,287	394 (7.5)	9.5	73.3	27.5	11.6	5.6	0	4.1

Women (n = 5,983 )	Prevalence^a^		Distribution of main reasons for discriminatory experience^b^ (%)						
	No. of respondents^c^	N (%)	sex	education	age	disability	birth region	others	not reply
Hired	4,062	721 (17.7)	36.9	41.9	45.9	1.9	1.1	5.4	0
Income	4,038	554 (13.7)	58.2	42.8	25.0	1.3	0.8	0	6.0
Training	3,574	64 (1.8)	49.1	43.6	23.6	3.6	0	0	14.1
Promotion	3,446	166 (4.8)** d	79.3	34.0	5.3	0.7	3.3	0	9.6
Fired	3,730	73 (2.0)	43.3	17.9	44.8	4.5	1.5	0	8.2
Education	5,086	178 (3.5)*** d	92.6	3.4	0.6	3.4	1.7	0	1.1
At home	5,543	344 (6.2)*** d	95.9	1.8	2.3	1.2	0	0	0.6
Societal activities	5,521	437 (7.9)	57.7	47.0	27.7	4.7	3.0	0	1.6

a Prevalence of perceived discriminatory experience in each situation.

b Since respondents are allowed to answer multiple causes, the sum of proportion in each situation can be added up to over 100%.

c The number of respondents for each questions after excluding the respondents who answered “Not applicable”.

d P-value: Chi-square test about the difference of prevalence of perceived discriminatory experience between men and women in each situation (Legend: * p<.05;** p<.01;

***p<.001).

In initial analyses, we tested effect modification by gender in the association between perceived discriminatory experience and poor self-rated health in each of eight situations. The p-value for the gender interaction term was not statistically significant except for discrimination at home. Accordingly, results are presented for the entire sample. Table 3 shows that the odds of being in poor health among individuals who reported experiencing discrimination is consistently higher, in unadjusted models, than that of those who reported no discrimination, with the exception of discrimination in promotion. Each cell in the table reflects a separate analytic model. When adjusted for demographic factors (age, gender and marital status), the odds ratios for work-related discrimination tend to get larger, those for discrimination in education and at home are substantially reduced and the odds for discrimination in social activities are essentially unchanged. With further adjustment for socio-economic status, the association between all types of discrimination and poor self-rated health is reduced but remains significant for discrimination in hiring, income, being fired and in general social activities

**Table 3:** Association Between Perceived Discriminatory Experience and Poor Self-Rated Health in Eight Situations, South Korea, 2004 (n = 11,544).

		Unadjusted OR		Adjusted for socio-demographic variables^a^		Adjusted for socio-demographics and SES^b^	
Situations	N^c^	OR	95% CI	OR	95% CI	OR	95% CI
Hired	8,455	1.47	(1.26, 1.72)	1.69	(1.42, 2.01)	1.34	(1.12, 1.61)
Income	8,462	1.38	(1.16, 1.64)	1.55	(1.28, 1.88)	1.33	(1.09, 1.62)
Training	7,689	1.56	(1.02, 2.37)	1.91	(1.17, 3.13)	1.61	(0.99, 2.62)
Promotion	7,470	0.89	(0.65, 1.22)	1.10	(0.78, 1.57)	1.20	(0.83, 1.74)
Fired	7,833	2.25	(1.59, 3.19)	1.84	(1.23, 2.76)	1.51	(1.01, 2.28)
Education	10,177	3.76	(2.77, 5.09)	1.61	(1.09, 2.38)	1.37	(0.94, 1.99)
At home	10,842	2.96	(2.38, 3.68)	1.37	(1.04, 1.79)	1.18	(0.90, 1.55)
Social activities	10,808	1.95	(1.64, 2.33)	1.97	(1.61, 2.41)	1.57	(1.28, 1.92)

a Adjusted for age, sex, marital status.

b Adjusted for age, sex, marital status, disability, income, education level, employment status including full-time student.

c Number of participants who answered Yes or No, not ‘Not Applicable’ in each situation and who are analyzed in the multiple logistic regression.

We also examined the extent to which there was a dose-response relationship between the number of situations of perceived discriminatory experiences and poor self-rated health (Table 4). After adjusting for potential confounding, the odds ratios for poor self-rated health for those exposed to one, two, three or four or more situations of perceived discrimination in their lifetime were respectively 1.06 (95% CI : 0.87–1.29), 1.15 (95% CI : 0.96–1.55), 1.59 (95% CI : 1.19–2.14) and 1.78 (95% CI:1.26–2.51).

**Table 4:** Association Between Perceived Discriminatory Experience and Poor Self-Rated Health, South Korea, 2004 (n = 11,544).

	Unadjusted		Adjusted for demographic variables		Adjusted for demographics and SES	
	OR	95% CI	OR	95% CI	OR	95% CI
No. of situations of discriminatory experience						
All NA	2.51	(2.03, 3.10)	1.88	(1.45, 2.43)	1.53	(1.19, 1.98)
0	1.00	Referent	1.00	Referent	1.00	Referent
1	1.49	(1.26, 1.76)	1.19	(0.99, 1.44)	1.06	(0.87, 1.29)
2	1.38	(1.13, 1.68)	1.42	(1.13, 1.79)	1.22	(0.96, 1.55)
3	1.87	(1.47, 2.37)	1.87	(1.41, 2.47)	1.59	(1.19, 2.14)
4 or more	1.84	(1.34, 2.52)	2.21	(1.55, 3.16)	1.78	(1.26, 2.51)
Age						
Continuous (years)			1.08	(1.08, 1.09)	1.04	(1.04, 1.05)
Gender						
Male			1.00	Referent	1.00	Referent
Female			1.49	(1.33, 1.67)	0.90	(0.79, 1.04)
Marital status						
Currently married			1.00	Referent	1.00	Referent
Never married			1.41	(1.11, 1.78)	1.14	(0.87, 1.48)
Previously married			1.25	(1.04, 1.49)	1.24	(1.04, 1.48)
Household income						
Less than 1Q					1.00	Referent
1Q–2Q					0.60	(0.51, 0.71)
2Q–3Q					0.47	(0.39, 0.56)
3Q-					0.37	(0.30, 0.45)
Education						
Junior high or less					1.00	Referent
High school					0.48	(0.41, 0.56)
College					0.29	(0.21, 0.41)
University and more					0.32	(0.25, 0.41)
Current employment status						
Unemployed					1.00	Referent
Full-time student					0.30	(0.19, 0.48)
Precarious employment					0.47	(0.37, 0.59)
Non-precarious employment					0.29	(0.23, 0.35)
Employer					0.46	(0.38, 0.56)

## Discussion

Although South Korea is widely viewed as a “one-ethnicity” country without racial discrimination, our findings suggest that multiple types of discrimination based on other social statuses occur in South Korea and that these self-reported experiences of discrimination were significantly associated with poor self-rated health. Perceived discriminatory experience in four of the eight situations was significantly related to poor self-rated health even after adjustment for demographic and socio-economic variables. And we also found a relationship between number of situations of perceived discriminatory experience and poor self-rated health, consistent with a dose-response relationship.

We did not find significant gender differences in the association between various types of perceived discrimination and poor-self rated health except for discrimination at home. This is different from a prior study of Asians in the U.S. found that women were more likely, compared to men, to have poor mental and physical health outcomes when they were exposed to a low level of discriminatory experience [38]. Future studies are needed to examine the extent to which gender may affect the association between discrimination and health outcomes in Korea, using more specific health outcome like schizophrenia or cardiovascular disease.

Regarding work-related discrimination such as discrimination in getting hired, there could be an issue about what constitute a discriminatory experience. It is possible that people report discriminatory experience when they did not get hired due to their educational level in a fair hiring process. But there are several reasons that our results may still be valid despite this possibility. First, it is well known that perceptions of discriminatory experience could play a role as a stressor and harm people's health regardless of the actual fairness of the hiring process [1]. Second, several studies have demonstrated the association between perceived discrimination and poor health outcome in prospective study designs after adjusting for potential psychological confounding factors [39]–[41]. Third, research reveals that respondents understand the concept of discrimination as intended by researchers and reports of personal experiences of discrimination are consistent with objective experiences [42], [43].

### Discrimination in South Korea

Our analyses found that their education level was the main cause of work-related discriminatory experiences in both men and women. In South Korea, education level is regarded as a type of “caste” because people's educational opportunities differ by socio-economic status, education level is critical in shaping one's life opportunities [44], [45], and education is an important contributor to wider inequalities in health [46], [47]. Even among university graduates, there is discrimination based on the university from which people graduated. For example, across different government administrations in South Korea, more than 45% of political leaders (ministers and vice ministers) since 1953, were graduates from one specific university [45]. This strong patterning of social opportunity could reflect both differences across universities in educational quality as well as, systemic preferences for graduates from selected universities.

Democratization and the feminist movement have sought to weaken the long tradition of patriarchy and the influence of Confucianism [22], [48] that has historically led to gender discrimination in South Korea. And in 1987, South Korea established the Equal Employment Act to prohibit gender discrimination in employment. However, consistent with evidence of a continuing substantial preference for sons that is responsible for gender-selective abortions [24], [25], the present study found that in 2004, South Korean women reported that they experienced various kinds of discrimination. A relatively high proportion of women indicated that they had been unfairly treated in getting hired and in receiving wages because of their gender, and compared to men, women reported a ten-fold higher prevalence of discrimination in getting higher education and at home. More than 90% of women reported their gender as the main reason for experiences of discrimination in the latter two situations.

Interestingly, South Korean men reported a higher prevalence of discrimination in promotion than women but this difference may be partially attributable to the reality of the long history of labor market discrimination that ensured that women had fewer opportunities to work outside the home compared to men [48], [49]. In addition, it is also possible that there are gender differences in the reporting of work discrimination, with women being more likely to deny personal experiences of work discrimination than men [50].

More attention also needs to be paid to age as an important source of discrimination in getting fired, for both men and women. South Korea is one of the world's fastest ageing societies with the lowest birth rate among the Organization for Economic Co-operation and Development (OECD) countries [51]. In post-hoc analyses in the group who reported discrimination in getting fired, 88% of those over 64 years old attributed their experience of this discriminatory experience to their age. In contrast, only 36% of respondents in under 65 years old viewed age as the reason for being unfairly fired. The labor force participation rate of people over 64 in South Korea is more than twice that of other OECD countries [52]. A primary reason for this high participation rate is that many older persons confront considerable financial distress if they do not continue to work, even if they often have to work in precarious or low paying jobs [53]. Given this situation, the health effect of unfair dismissal for the elderly in South Korea can have a large negative impact on their economic well-being as well as their health. Thus, the recent enactment of the “Age Discrimination in Employment Act” in 2010 in South Korea has the potential to enhance the health of the elderly.

Consistent with the view that South Korea is an ethnically homogeneous nation, our analyses did not find substantial reports of racial discrimination. However, there are people with “mixed-blood” in South Korea who have experienced discrimination in their daily lives in a South Korea society which gives preference to those who have a “pure-bloodline” [54]. Additionally, there are a growing number of foreign residents including both workers and students, coming mainly from China, the US, the Philippines, Japan and Vietnam in South Korea. They currently account for slightly more than two percent of the total population [55] but they are likely to be a higher proportion in the future. Research reveals that many of these individuals are exposed to pervasive discrimination in their daily lives, particularly migrant workers who have to face employment discrimination, economic exploitation and appalling working conditions [56], [57]. These populations, because of their small size, are not present in large numbers in a typical national sample but future targeted studies should he levels and consequences of discrimination in these stigmatized groups.

### Limitations and strengths

The limitations of the study include the validity of the measure used to assess discrimination. The “Experience of Discrimination” questionnaire was developed to measure exposure to racial or gender discrimination in the U.S., and it is known to have reasonable psychometric properties in U.S. studies [28], [58]. However, its validity for the South Korean context is unknown. For example, improving the discrimination questionnaire to be more sensitive to the social context in South Korea might require questions about discriminatory experience at home or at general social activities to be more specific to assess discrimination accurately. Questions about discriminatory experiences at home could usefully clarify whether the experiences originated with parents, a spouse or a significant other. Further studies are necessary to assess the cross-cultural validity and reliability of discrimination questions in South Korea. In addition, one version of the “Experience of Discrimination” questionnaire includes information about the frequency of discriminatory experiences in each situation [58], but the version used in the present study assessed only whether respondents had experienced discrimination in their lifetime.

Second, because of the cross-sectional nature of the study, we are unable to identify any temporal ordering among the associations between perceived discrimination and health. Thus we are unable to rule out the possibility of reverse causation in which people in poor health would be more likely to report experiences of discrimination. This issue has been addressed in the larger literature on discrimination and health and prior research has documented that reports of discrimination are often associated with subsequent changes in physical and mental health [1].

A major strength of this study is its large sample size from a nationally representative sample of the South Korean population. Whereas previous studies have focused on the health effects of racial discrimination in Western countries, we found that non-racial discriminatory experiences are associated with poor health outcomes in an Asian country as well. Secondly, this study founds a consistent association between perceived discriminatory experience and health across multiple situations after adjusting for potential confounding by gender, age, education level and household income.

To our knowledge, this is the first nationally representative study conducted in an Asian country that explored the association between perceived discrimination and health. It provides a glimpse of respondents' reports of the prevalence of multiple forms of non-racial discrimination and documents that these experiences are positively related to poor self-rated health. These findings, if replicated, suggests that experiences of discrimination may be an important determinant of health and of health disparities in South Korea and other Asian countries.
